# *Treponema denticola* Major Outer Sheath Protein Impairs the Cellular Phosphoinositide Balance That Regulates Neutrophil Chemotaxis

**DOI:** 10.1371/journal.pone.0066209

**Published:** 2013-06-03

**Authors:** Michelle B. Visser, Chun-Xiang Sun, Adeline Koh, Richard P. Ellen, Michael Glogauer

**Affiliations:** Matrix Dynamics Group, Dental Research Institute, University of Toronto, Toronto, Ontario, Canada; Cardiff University, United Kingdom

## Abstract

The major outer sheath protein (Msp) of *Treponema denticola* inhibits neutrophil polarization and directed chemotaxis together with actin dynamics *in vitro* in response to the chemoattractant *N*-formyl-methionine-leucine-phenylanine (fMLP). Msp disorients chemotaxis through inhibition of a Rac1-dependent signaling pathway, but the upstream mechanisms are unknown. We challenged murine bone marrow neutrophils with enriched native Msp to determine the role of phospholipid modifying enzymes in chemotaxis and actin assembly downstream of fMLP-stimulation. Msp modulated cellular phosphoinositide levels through inhibition of phosphatidylinositol 3-kinase (PI3-kinase) together with activation of the lipid phosphatase, phosphatase and tensin homolog deleted on chromosome 10 (PTEN). Impaired phosphatidylinositol[(3,4,5)]-triphosphate (PIP3) levels prevented recruitment and activation of the downstream mediator Akt. Release of the actin capping proteins gelsolin and CapZ in response to fMLP was also inhibited by Msp exposure. Chemical inhibition of PTEN restored PIP3 signaling, as measured by Akt activation, Rac1 activation, actin uncapping, neutrophil polarization and chemotaxis in response to fMLP-stimulation, even in the presence of Msp. Transduction with active Rac1 also restored fMLP-mediated actin uncapping, suggesting that Msp acts at the level of PIP3 in the hierarchical feedback loop of PIP3 and Rac1 activation. Taken together, Msp alters the phosphoinositide balance in neutrophils, impairing the cell “compass”, which leads to inhibition of downstream chemotactic events.

## Introduction

Neutrophils are key rapid-response cells of the innate immune system that are recruited to sites of infection to eradicate pathogens [1]. Neutrophils migrate in a directed manner in response to chemoattractants such as cytokines, complement peptides, bacterial products and chemicals including peptides bearing the *N*-formyl group (*N*-formyl-methionine-leucine-phenylanine, fMLP). Key to chemotaxis is the ability of neutrophils to polarize, with formation of a leading edge (lamellipodium) toward the chemoattractant and a trailing edge (uropod). During chemotaxis, movement of the cell is mediated by dynamic protrusion and retraction of the lamellipodium and uropod, respectively, driven by pathways that regulate remodeling of the actin cytoskeleton [2], [3]. Polarization and chemotaxis require asymetrical distribution of molecules within the cell; including the accumulation of phosphatidylinositol[(3], [4], [5)]-triphosphate (PtdIns [(3], [4], [5)]P_3_, PIP3) at the leading edge [4], [5]. Proper spatial localization and accumulation of PIP3 has been proposed to act as an internal cellular “compass”, leading to efficient directional migration [6], [7], [8].

Production of PIP3 is catalyzed from phosphatidylinositol 4,5-bisphosphate (PtdIns [(4], [5)]P_2_) by the lipid kinase phosphatidylinositol 3-kinase (PI3-kinase) [9]. PIP3 mediates cellular functions, including cell polarity, by recruiting proteins containing pleckstrin homology domains as well as activating small GTPases, leading to localized cytoskeleton rearrangement at the leading edge [10], [11]. PIP3 levels are counteracted by two lipid phosphatases; the 3′ phosphatase and tensin homolog (PTEN) and the 5′ SH2-containing inositol phosphatase1 (SHIP1), which generate PtdIns[(4], [5)]P_2_ and PtdIns[(3], [4)]P_2_, respectively [12], [13]. Numerous studies using chemical inhibitors and gene knock-out mice have implicated complex interconnecting roles of PI3-kinase, PTEN and SHIP1 in the regulation of neutrophil directional sensing and migration [14], [15], [16], [17], [18], [19], [20].

*Treponema denticola* is a key pathogen in the polymicrobial infection associated with chronic periodontitis [21], [22]. The major outer sheath protein (Msp) is expressed as a high- molecular weight complex in the outer sheath (outer membrane) of *T. denticola* as well as in shed vesicles [23], [24], [25]. Msp is considered to be one of *T. denticola*'s major virulence factors. It interacts with multiple host proteins and cells leading to changes in host cell homeostasis (for review see [26], [27]). Serum antibodies to Msp are present in humans with gingival inflammation, confirming that this spirochete surface protein contacts and activates cells of the immune system [28].

Msp is well known for perturbing signaling pathways that regulate the cytoskeletal dynamics of host cells, including neutrophils [29], [30], [31]. It has been reported to selectively inhibit Rac1 activation and impair spatially localized accumulation of Rac1 and PI3-kinase products downstream of fMLP stimulation [30], key processes required for effective neutrophil chemotaxis. Yet, the underlying mechanisms are poorly understood. Here we investigate the contribution of phosphoinositide modifying enzymes and their impact on PIP3 related Msp-mediated defects in chemotaxis and actin dynamics downstream of chemoattractant activation in neutrophils.

## Materials and Methods

### Ethics statement

Mice were used in accordance with the Guide for the Humane Use and Care of Laboratory Animals and approval of the University of Toronto animal care committee (Protocol number 20009508). Mice were euthanized by CO_2_ followed by cervical dislocation.

### Treponema denticola Msp enrichment

Native Msp complex was enriched from New Oral Spirochete broth cultures of *T. denticola* ATCC 35405 as previously described [32], [33]. Msp was used at a concentration of 6 µg/ml, a standard concentration which has been shown to impair Rac1 activation and actin dynamics in neutrophils [30], [31]. All experiments were performed with Msp pre-treatment for 30 minutes prior to stimulation with 1 µM fMLP (Sigma) for 1 minute.

### Murine neutrophil preparation

C57BL/6J wild-type mice (male, 6 weeks old) were purchased from Charles River. Femurs and tibias were removed and cells were isolated from bone marrow by fractionation into discontinuous Percoll (Sigma) gradients (80%, 65%, 55%). Mature neutrophils were isolated from the 80%/65% interface.

### Lipid phosphatase activity analysis

Lipid phosphatase activity was measured as described previously [6], [34]. Briefly, cells were partially permeabilized with 0.2% octyl glucoside (OG) prior to treatment with 150 nM VO-OHpic (Sigma) and/or Msp, as indicated, followed by stimulation with fMLP. Samples were incubated with the PTEN substrate, 3-methylenephosphonate diC8 (Echelon), and the amount of free phosphate released was measured using a malachite green phosphatase assay kit according to manufacturer's protocol (Echelon).

PTEN activity was determined using a PTEN immunoprecipitation assay. 5×10[6] neutrophils per condition were treated as indicated, lysed with lysis buffer (25 mM Tris pH 8.0, 150 mM NaCl, 1% Triton, 1 mM EDTA, 5% Glycerol), and immunoprecipitated using an anti-PTEN antibody (Cell Signaling, clone D4.3 XP) overnight at 4°C, followed by protein A agarose beads for 1 hour (Sigma). Beads with immunoprecipitated PTEN were washed three times with TBS with 10 mM DTT, followed by incubation with 3000 pmol of soluble PtdIns[(3], [4], [5)]P_3_ substrate for 1 hour at 37°C (Echelon). The amount of free phosphate released due to conversion by PTEN was determined using Malachite Green solution (Echelon).

### PI3-kinase activity analysis

PI3-kinase activity was measured using a PI3-Kinase activity ELISA: Pico (Echelon) according to the manufacturer's protocol. Briefly, 5×10[6] cells were treated per condition, and reactions terminated by lysis in buffer (20 mM Tris pH 7.4, 1% Triton, 137 mM NaCl, 1 mM CaCl2, 1 mM MgCl2, 0.1 mM sodium orthovanadate, 1 mM PMSF). Supernatant was mixed with an anti-PI3-kinase antibody overnight at 4°C (p85, Millipore), followed by protein A agarose beads for 1 hour. Immunoprecipitated PI3-kinase was incubated with PtdIns[(4], [5)]P2 substrate for 2.5 hours at 37°C and the amount of PtdIns[(3], [4], [5)]P_2_ produced was determined using a competitive ELISA assay (Echelon).

### Akt analysis

Neutrophils were treated, followed by fMLP stimulation, lysed in 5X SDS sample buffer, and boiled for 10 minutes. Lysates were separated by SDS- PAGE followed by transfer to nitrocellulose and membranes were blocked with 5% skim milk/ TBS/ 0.05% Tween-20 and incubated overnight with anti-Phospho-Akt antibody (1∶1000, Cell signaling Technology). Membranes were incubated with HRP-linked secondary antibody followed by detection using the Western Lightning ECL substrate (Perkin-Elmer). Membranes were re-probed with a total Akt antibody (1∶1000, Cell Signaling Technology).

For immunofluorescence analysis, Msp-treated neutrophils were allowed to attach to BSA-coated coverslips followed by stimulation with fMLP. Cells were fixed with 4% paraformaldehyde for 10 minutes, permeabilized with 0.5% Triton X-100 for 15 minutes and blocked with 5% normal goat serum (NGS) for 30 minutes. Anti-Akt antibody (Cell Signaling Technology) was diluted in 1∶50 in 5% NGS and applied for 1 hour at room temperature followed by Alex-488 goat anti- rabbit secondary antibody (1∶100, Invitrogen) for 1 hour. F-actin was labeled with Alexa-594 phalloidin (1∶40, Invitrogen) and nuclei stained with 4′,6-diamidino-2-phenylindole (DAPI, Roche). Cells were examined by epifluorescence microscopy (Leica DMIRE2) and images obtained using OpenLab softwatre (Perkin Elmer).

### Actin capping protein quantification

The actin capping proteins gelsolin and CapZ were analyzed as described [35]. Neutrophils were partially permeabilized with 0.2% OG buffer prior to treatment, as indicated, followed by fMLP stimulation. A portion of the supernatant was collected for analysis of released protein while the remaining cells were collected and lysed in SDS sample buffer. Samples were analyzed by SDS-PAGE and western blotting for gelsolin (1∶2000 Rabbit polyclonal antibody, CAG McCulloch, University of Toronto) or capZ (1: 100, mAb 5B12.3, Developmental Studies Hybridoma Bank).

### TAT-protein transduction

The Rac1 constitutively active (Rac1CA, G12V) and empty TAT-HA constructs were provided by Dr. Gary Bokoch (Scripps Research Institute, La Jolla, CA). Protein expression and purification was performed as described [6] with slight modification. Briefly, BL21 (DE3)-pLysS transformed cultures were inoculated 1∶100 with overnight culture, grown 6 hours at 37°C, followed by induction for 2 hours with 1 mM IPTG. Cell pellets were resuspended in lysis buffer (7M Urea, 100 mM NaH_2_PO_4_, 10 mM Tris pH 8.0, 5 mM imidazole), treated with benzonase (3 U/ml, Sigma), and sonicated (4×20 seconds). Samples were centrifuged and His-Tag-TAT proteins purified from supernatants using Ni-NTA superflow columns (Qiagen) according to manufacturer's protocol for denaturing conditions, except proteins were eluted from the column with 8M Urea, 100 mM NaH_2_PO_4_, 10 mM Tris pH 8.0, 0.5M imidazole. Samples were dialyzed using 15 ml Slide-a-Lyzer dialysis cassettes (3000 MWCO, Pierce) overnight with 2 changes of buffer against PBS/ 5% glycerol followed by concentration with Amicon centrifugation devices (3000 MWCO, Millipore).

To determine the effect of Rac1CA or TAT-HA rescue on actin capping protein release, neutrophils were transduced with 500 nM purified TAT-protein for 10 minutes following OG permeabilization prior to Msp treatment and actin uncapping quantification as described above.

### Chemotaxis and polarization analysis

Cells were treated as indicated prior to being resuspended in Hank's Balanced Salt Solution (HBSS), pH 7.4, with 1% gelatin and allowed to attach to 1% BSA coated coverslips (22×40 mm) at 37°C for 10 minutes. Coverslips were inverted onto a Zigmond chamber, and HBSS solution was added to the left chamber while fMLP was added to the right chamber. Images were taken every 20 seconds for 15 minutes using a Nikon Eclipse microscope E1000 equipped with a Hamatsu camera (model ORCA-ER). Images were analyzed using ImageJ software and chemotaxis analysis (migration and speed) performed using the manual tracking and chemotaxis tools in Image J. The number of cells polarized following 5 minute exposure to fMLP was calculated from still images obtained during the chemotaxis assay [30].

### Rac1 activation assay

The amount of active Rac1 was determined using a PAK-PBD pulldown assay. Treated neutrophils were lysed in 2X lysis buffer (20 mM HEPES pH 7.4, 150 mM NaCl, 2% Triton, 20% glycerol, 8 mM EDTA, 8 mM EGTA, 2X protease inhibitor cocktail (Sigma)) and PAK-PBD-GST beads (20 µg, Cytoskeleton Inc) were added to the lysates for 1 hour at 4°C, followed by collection. Bead samples were washed two times (20 mM HEPES pH 7.4, 1% Triton, 150 mM NaCl, 10% glycerol, 4 mM EDTA, 4 mM EGTA), followed by resuspension in 5X SDS sample buffer. Bead samples and total lysates were analyzed by SDS- PAGE and western blotting with a Rac1 antibody (1∶1000, Upstate).

### Statistical analysis

Comparisons between two groups were performed using t-tests. Results are based on at least 3 independent experiments. Statistical significance was defined as *P*<0.05.

## Results

### Msp activates lipid phosphatase activity in neutrophils

**Figure 1:**
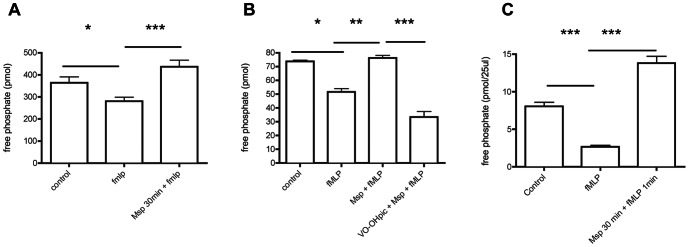
Msp activates lipid phosphatase activity, including PTEN. A) Pretreatment of neutrophils with Msp increases lipid phosphatase activity measured in partially permeabilized cells using a malachite green assay. B) Treatment of neutrophils with the bisperoxovanadate compound VO-OHpic, a PTEN inhibitor, prevented Msp-mediated phosphate release. C) Immunoprecipitated PTEN from cell lysates was used to measure specific PTEN activity following Msp pretreatment prior to fMLP stimulation. For these assays, activity was measured by incubation with soluble diC8 PIP3, and phosphate release was measured using the malachite green assay. Graphs represent the mean ± SEM of 3 independent experiments (* P<0.05, ** P<0.01, *** P<0.001 unpaired t test).

Cellular phosphoinositide levels are tightly regulated through the actions of both PI3-kinases and phosphoinositide phosphatases. Lipid phosphatase activity was assessed in partially permeabilized neutrophils using a malachite green assay to measure dephosphorylation of a synthetic PIP3 substrate. Pretreatment of neutrophils with Msp prior to fMLP stimulation increased the release of free phosphate compared with fMLP stimulation alone, indicating activation of a lipid phosphatase (Figure 1A). To determine the lipid phosphatase involved, the bisperoxovandate compound VO-OHpic, a PTEN inhibitor [36], was used. Pretreatment with VO-OHpic prevented Msp-mediated phosphatase activity in response to fMLP stimulation (Figure 1B). To confirm these results, we used a specific PTEN immunoprecipitation assay. PTEN was immunoprecipitated from treated neutrophils and the amount of PIP3 converted to PIP2 was measured using a malachite green phosphatase assay. Msp pretreatment prior to fMLP stimulation increased the PTEN activity relative to fMLP stimulation alone (Figure 1C). In these experiments, Msp treatment alone also increased PTEN activity by approximately 40% (data not shown, P<0.05 by unpaired t test compared to control). Together these results indicate that Msp is able to stimulate PTEN activity in neutrophils and that exposure to Msp leads to PIP3 to PIP2 conversion in both resting and fMLP-activated cells.

### Msp inhibits PI3-kinase activity and PIP3 production

**Figure 2:**
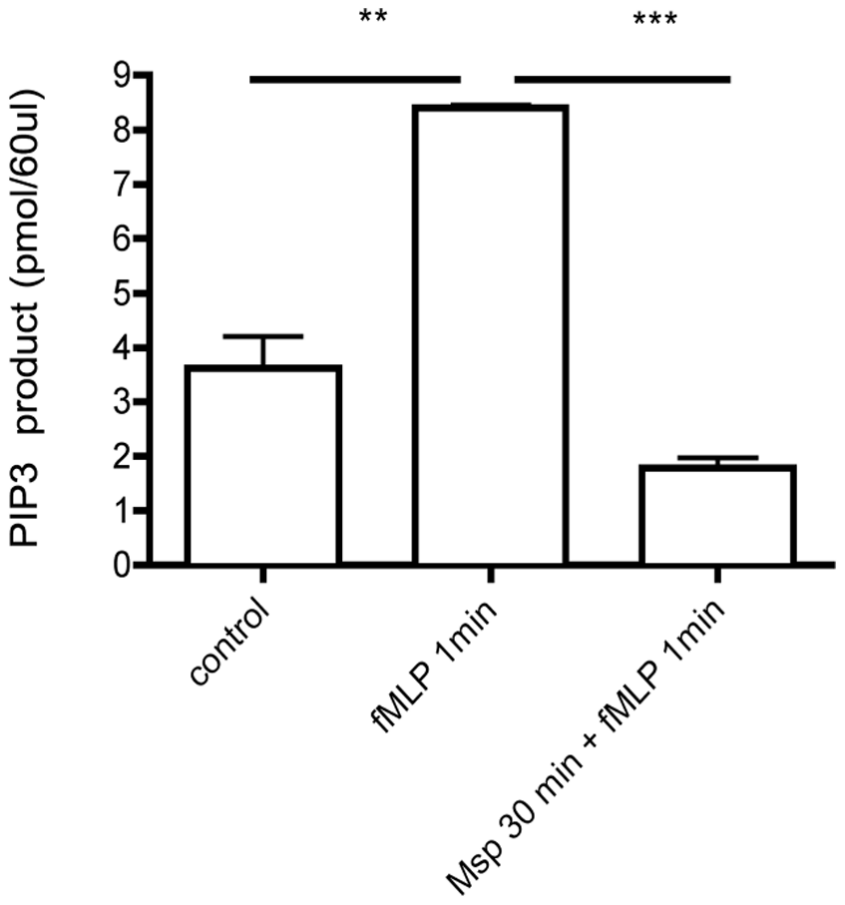
Msp inhibits PI3-kinase activity in response to fMLP stimulation. Neutrophils were treated as indicated, lysed, and PI3-kinase was immunoprecipitated. Immunoprecipitated PI3-kinase was incubated with PIP[(4], [5)]2 substrate and the amount of resultant PIP3 was measured by ELISA. Graph represents the mean ± SEM of 3 independent experiments ( ** P<0.01, *** P<0.001, unpaired t test).

As Msp is able to inhibit Rac1 activation selectively and the polarized accumulation of PI3-kinase products in fMLP-activated neutrophils [30], we examined the effect of Msp on PI3-kinase activity following fMLP stimulation. Exposure to fMLP alone significantly increased the amount of PIP3 produced by immunoprecipitated PI3-kinase, whereas Msp pretreatment yielded significantly decreased PIP3 production following fMLP stimulation (Figure 2).

**Figure 3:**
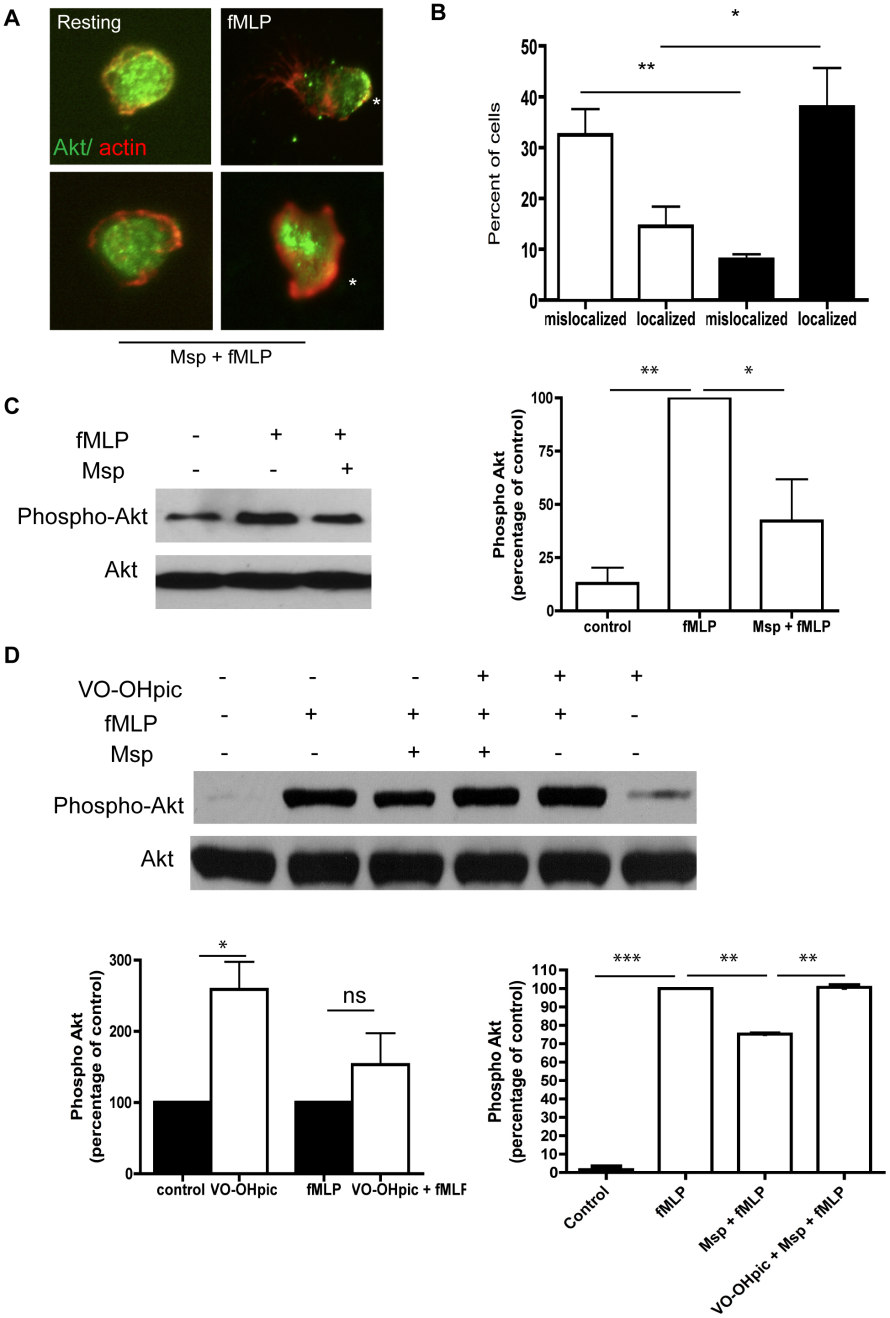
Msp impairs Akt localization and activation upon fMLP stimulation. Akt is a downstream target of PI3-kinase. Akt localization and activation can be considered indirect measures of PIP3 generation. A) Localization of Akt and actin in neutrophils visualized by indirect immunofluorescence. Alexa 594-phalloidin F-actin label in red and Akt antibody label in green. Asterix indicates leading edge of neutrophil. B) Graph represents the percentage of cells in which Akt and actin fluorescence are localized together or mislocalized (mean ± SEM, Msp + fMLP treatment (white bars), fMLP treatment alone (black bars). C) Phosphorylation of Akt in cell lysates measured by immunoblotting, following Msp pretreatment and fMLP stimulation. D) PTEN inhibition with the inhibitor VO-OHpic increased PIP3 production and downstream signaling (left panel graph). Treatment of neutrophils with VO-OHpic prior to Msp treatment restored PIP3 production and Akt signaling in response to fMLP stimulation (right panel graph). Representative phospho-Akt immunoblots are shown in panels B and C. Graphs represent the mean ± SEM densitometry of 3 independent experiments (* P<0.05, ** P<0.01, *** P<0.001, ns =  not significant, unpaired t test).

Akt is a downstream target of PI3-kinase; its recruitment and activation can be considered an indicator of PIP3 generation. PIP3 binds Akt and recruits it to the plasma membrane, where Akt is activated by phosphorylation. Immunofluorescence analysis of fMLP-stimulated PMNs revealed re-localization of Akt (Figure 3A, green) from the cytoplasm to the near-membrane actin-rich lamellipodium (Figure 3A, red). Msp pretreatment prevented the re-localization of Akt to the plasma membrane, with primarily diffuse cytoplasmic localization similar to that of resting control cells (Figure 3A, lower left panel). Notably, in cells in which partial polarization was observed, Akt was still localized at the center or rear of the cell (Figure 3A, lower right panel). In neutrophils stimulated with fMLP alone, significant co-localization of actin and Akt immunostaining was observed, whereas Msp pretreatment decreased the percentage of cells that demonstrated localization of these molecules (Figure 3B). Downstream activation of Akt, measured by western analysis of Akt phsophorylation in cell lysates, was also evaluated following Msp treatment. Stimulation of neutrophils with fMLP alone resulted in a robust increase in Akt phosphorylation, whereas pretreatment with Msp prior to fMLP stimulation prevented Akt phosphorylation (Figure 3C, D).

Inhibition of PTEN with the inhibitor VO-OHpic increased the PIP3 levels in neutrophils, as measured indirectly through the impact on Akt phosphorylation levels (Figure 3D). When PIP3 production and signaling were restored by pretreatment of the neutrophils with VO-OHpic, the cells overcame impairment of Akt phsophorylation by Msp in response to fMLP (Figure 3D). Together, these results indicate that Msp impairs PIP3 production and downstream signaling pathways in response to fMLP stimulation by modulating the PI3-kinase/PTEN pathways.

### Mspinhibits actin uncapping in response to fMLP

**Figure 4:**
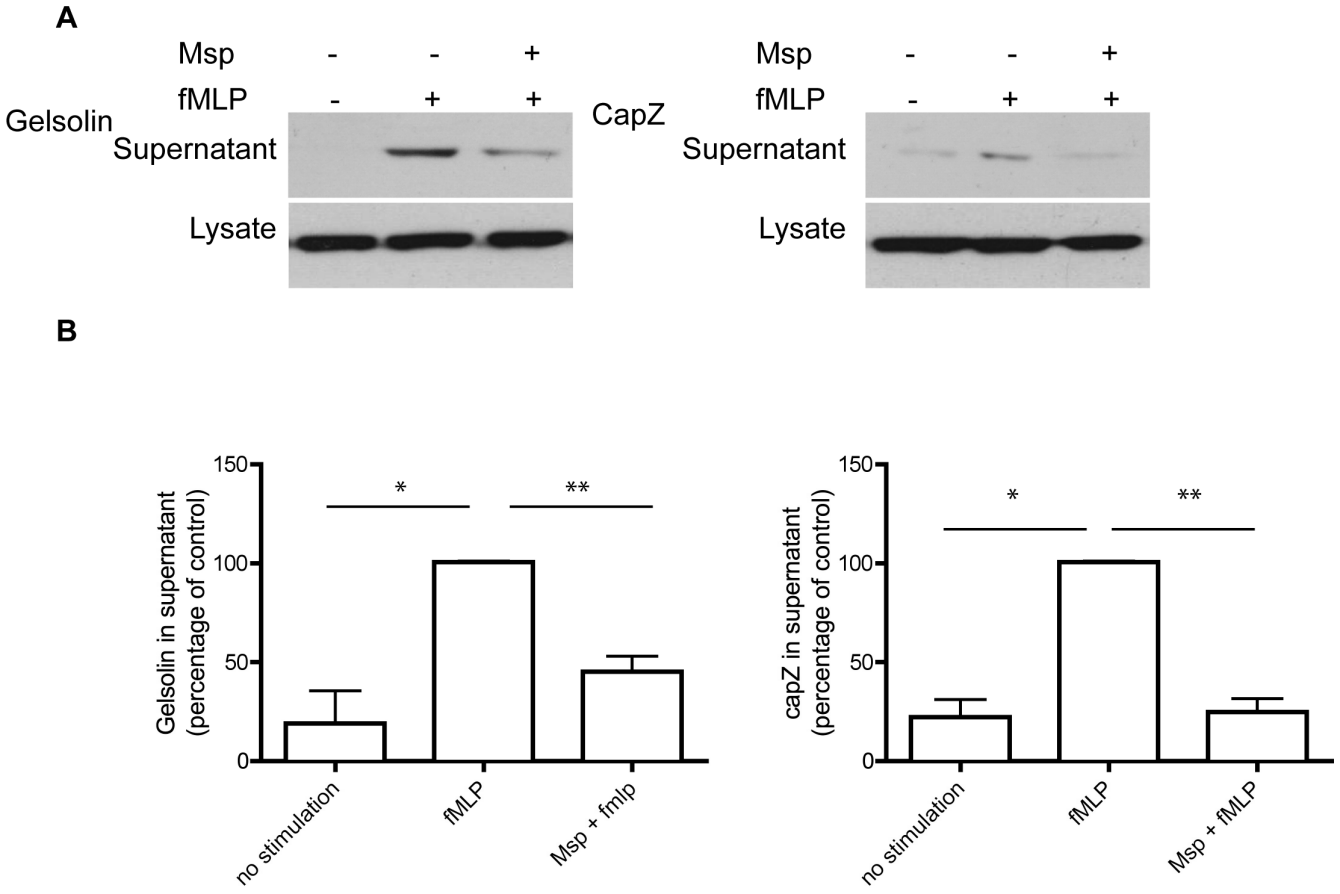
Msp impairs actin uncapping in response to fMLP stimulation. A) Immunoblotting of partially permeabilized neutrophils was used to quantify release of the actin capping proteins gelsolin (Left) and capZ (right). Cells were partially permeabilized prior to Msp treatment followed by fMLP stimulation, and released protein supernatants were analyzed. Representative immunoblots are shown. B) Graphs of densitometry analysis of immunoblots represent mean ± SEM (n = 3, * P<0.05, ** P<0.01 unpaired t test).

The balance of membrane phosphoinositides in the cell regulates several functions, including actin rearrangement and subsequent locomotion. As Msp has been found to impair actin free barbed end formation in response to fMLP in neutrophils [31] and impact the release of actin capping proteins in fibroblasts [34], we chose to investigate actin uncapping in neutrophils following Msp treatment. To assess actin uncapping, we performed western immunoblotting on supernatants of partially permeabilized neutrophils to quantify capping proteins gelsolin and capZ released from the cell. Control cells stimulated with fMLP showed increased release of both gelsolin and capZ, whereas Msp pretreatment prevented the release of these capping proteins from the actin filament ends in response to fMLP (Figure 4), indicating more avid association of the capping proteins with actin filaments following Msp treatment.

**Figure 5:**
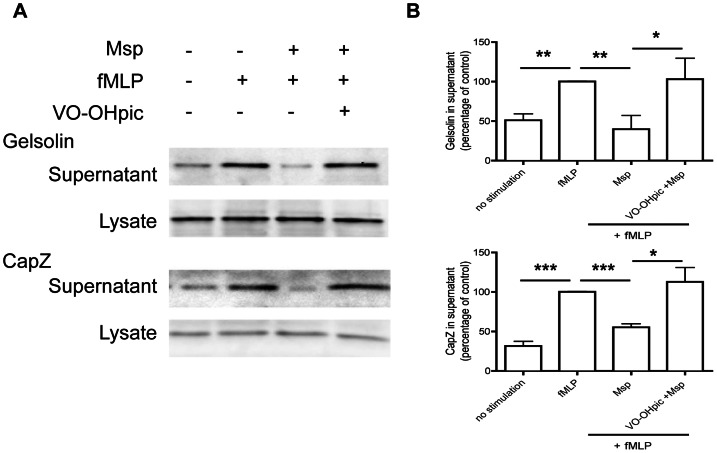
PTEN inhibition restores Msp mediated impairment of actin uncapping. A) Partially permeabilized neutrophils were treated with VO-OHpic prior to Msp treatment followed by fMLP stimulation. The amount of gelsolin and capZ released from actin filaments was quantified in collected supernatants and cell lysates by immunoblotting. Representative immunoblots are shown. B) Graphs of densitometry analysis of immunoblots represent mean ± SEM (n = 3, * P<0.05, ** P<0.01, *** P<0.001, unpaired t test).

PTEN inhibition in neutrophils increased PIP3 levels (Figure 3D,[17], [36]), leading to increased Akt activation (Figure 3D, [37]) and increased actin polymerization [20]. Therefore, we also determined the effect of PTEN inhibition on Msp-mediated actin assembly defects. Chemical inhibition of PTEN activity in neutrophils prior to Msp treatment restored the release of the actin capping proteins gelsolin and capZ downstream of fMLP stimulation compared with Msp treatment alone (Figure 5). Thus, by restoring PIP3 levels through PTEN inhibition, we rescued Msp-mediated impairment of actin dynamics subsequent to fMLP stimulation.

**Figure 6:**
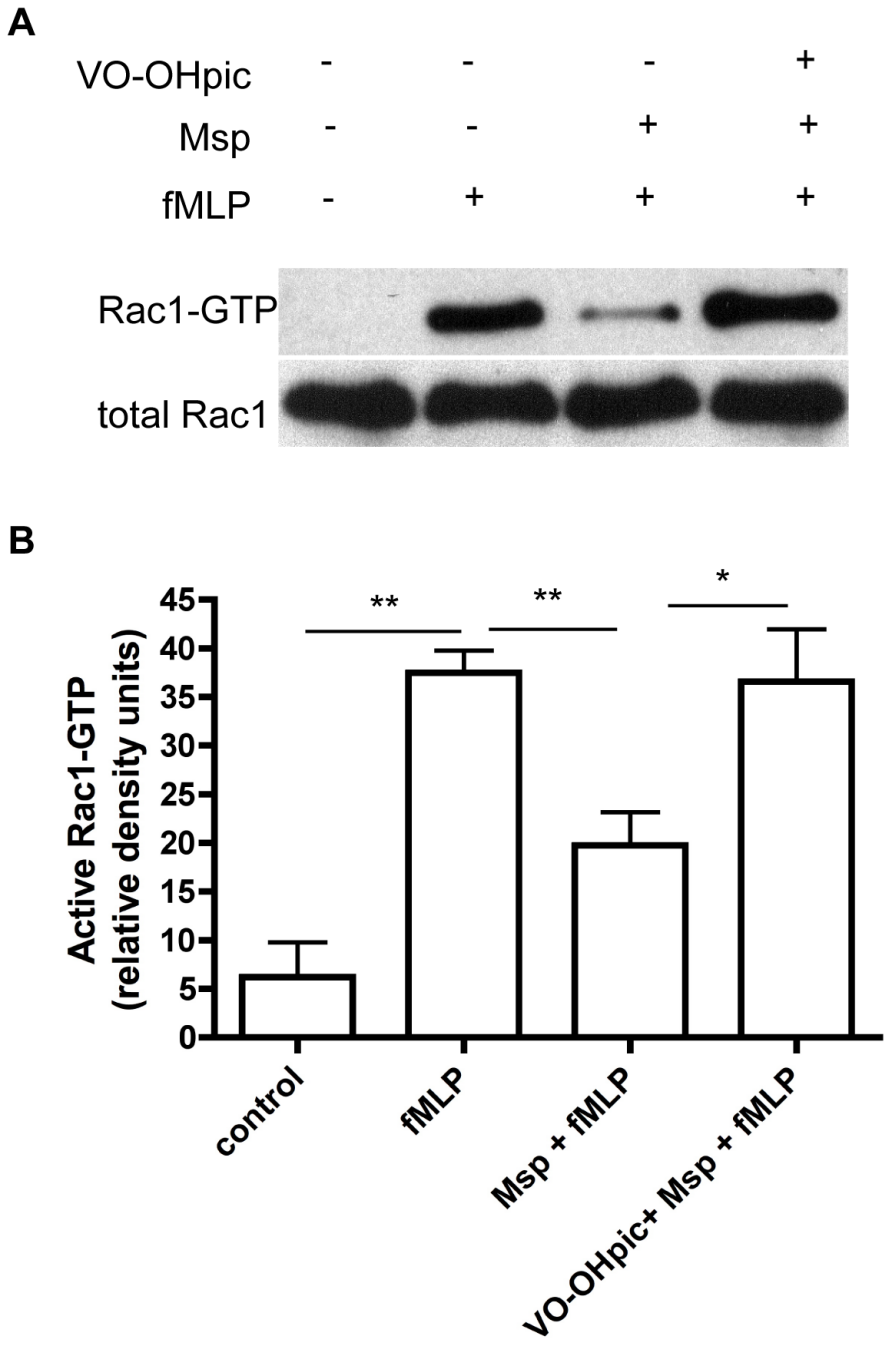
PTEN inhibition restores Msp-mediated Rac1 activation. PAK-PBD pulldown assays were used to quantify active Rac1 in neutrophils in response to fMLP. Msp-mediated Rac1 inhibition in response to fMLP was reversed by treatment of cells with VO-OHpic. A) A representative immunoblot of active Rac1 quantification is shown. B) Graph of densitometry analysis represents mean ± SEM (n = 3, * P<0.05, ** P<0.01, unpaired t test).

Since PTEN inhibition restored ‘physiological’ PIP3 signaling in Msp-treated neutrophils, we also determined its impact on the previously recognized Msp-mediated inhibition of Rac1 activation in fMLP-stimulated cells [30]. As anticipated, Msp pretreatment of neutrophils reduced Rac1 activation in response to fMLP compared with fMLP stimulation alone (Figure 6, [30]). Rac1 activity was restored by chemical inhibition of PTEN (Figure 6). Thus, restoration of PIP3 signaling also restored Rac1 activation by fMLP, even in the presence of Msp.

**Figure 7:**
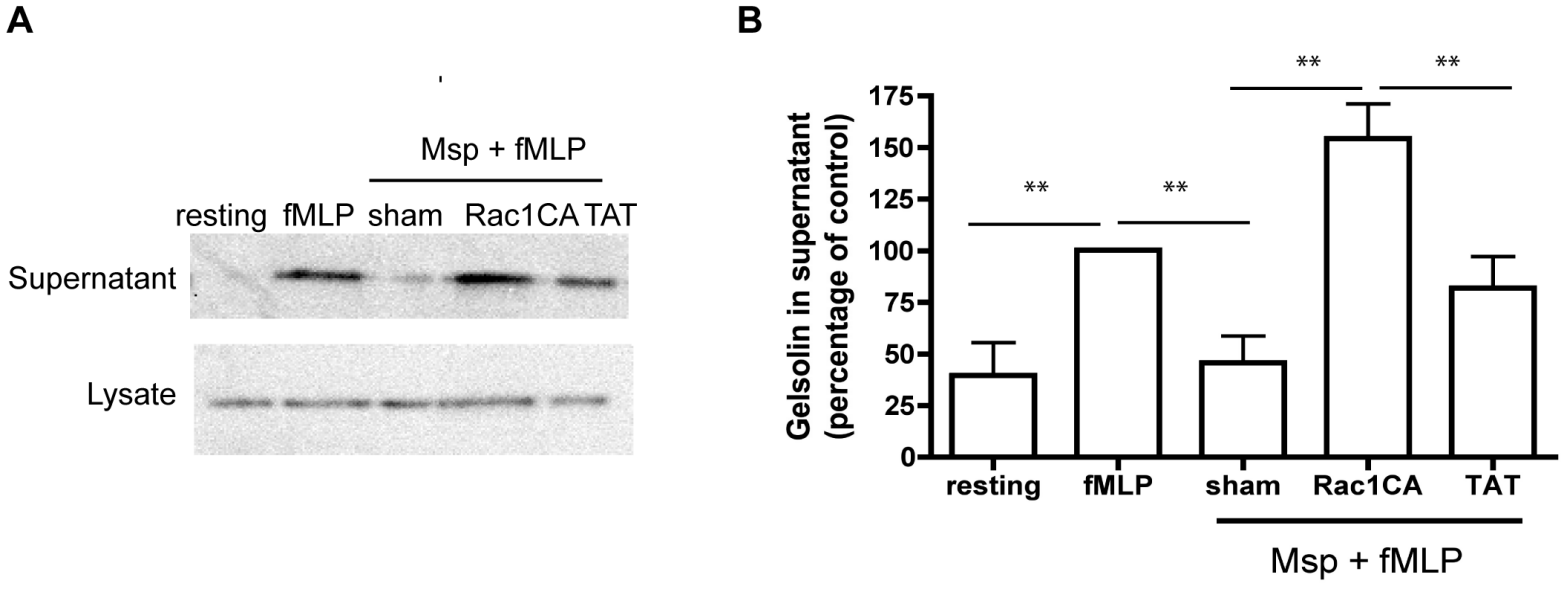
Rescue of active Rac1 is able to restore Msp mediated defects in actin uncapping. TAT-mediated transduction of active Rac1 (Rac1CA) was able to rescue Msp-mediated defective gelsolin uncapping in response to fMLP stimulation. Cells were partially permeabilized followed by protein transduction with either 500 nM Rac1CA-TAT protein or TAT protein as a control, prior to Msp treatment and fMLP stimulation. A) A representative immunoblot showing the amount of gelsolin released following Msp treatment and fMLP stimulation. B) Graph represents densitometry analysis mean ± SEM (n = 4, ** P<0.01, unpaired t test).

Rac1 is the dominant mediator of actin filament uncapping downstream of the fMLP receptor [35]. We determined the effect of active Rac1 overexpression on actin uncapping in Msp-treated neutrophils. Transduction of neutrophils with constitutively active TAT-Rac1G12V (Rac1CA) protein, which is not dependent on upstream signaling, prior to Msp treatment restored the release of the actin capping protein gelsolin in response to fMLP stimulation compared with neutrophils treated only with Msp or to neutrophils transduced with a control TAT-HA protein prior to fMLP stimulation (Figure 7). This suggests that rescue of active Rac1, which is normally inhibited by Msp exposure, restores normal actin dynamics upon fMLP stimulation in neutrophils.

### PTEN inhibition restores Msp-mediated defects in chemotaxis

**Figure 8:**
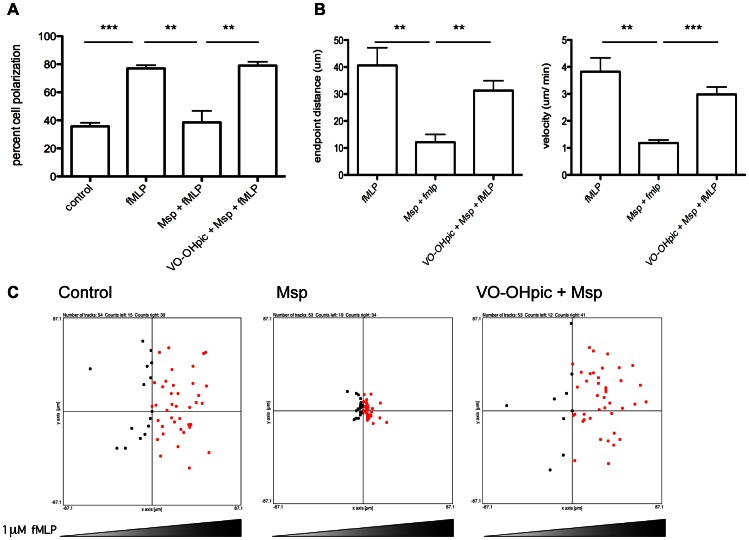
PTEN inhibition restores Msp-mediated defects in neutrophil polarization and chemotaxis. Neutrophil migration toward fMLP was assessed using Zigmond chambers. Neutrophils were treated with VO-OHpic and Msp as indicated, attached to coverslips and exposed to an fMLP gradient for 10 minutes. A) The percentage of cells which displayed polarized morphology is shown. B) The total endpoint distance migrated and the velocity of migration is shown. Graphs represent mean ± SEM of 3 independent experiments (** P<0.01, *** P<0.001, unpaired t test). C) Representative plots of cell endpoint location with respect to the origin are shown. Approximately 50 cells were counted per condition.

Accumulation of PIP3 at the leading edge of neutrophils is crucial for the regulation of directed migration in response to fMLP through recruitment and activation of secondary mediators and local actin polymerization. Msp inhibits both neutrophil polarization and chemotaxis [30]. As PTEN inhibition restored the typical actin dynamics in response to fMLP following Msp treatment, we next asked whether decreased PTEN activity would reverse Msp inhibition of neutrophil polarization and chemotaxis. Chemotaxis in an fMLP gradient was analyzed using Zigmond chambers. As neutrophil polarization is a prerequisite to chemotaxis, we also examined the percentage of cells that assumed a polarized shape. As previously reported [30], Msp prevented effective neutrophil polarization in response to fMLP. In contrast, chemical inhibition of PTEN reversed this effect (Figure 8A). Neutrophils pretreated with Msp alone were unable to migrate effectively toward fMLP (Figure 8,[30]). However, neutrophils treated with the PTEN inhibitor VO-OHpic prior to Msp exposure migrated toward fMLP similarly to untreated control cells (Figure 8). Likewise, treatment with VO-OHpic restored, to control values, the neutrophil velocity and migration distance in the fMLP gradient, which were inhibited by Msp exposure alone (Figure 8).

## Discussion

Signaling and regulatory pathways that include plasma membrane-associated phosphoinositides, lipid kinases, and lipid phosphatases are crucial for determining the actin dynamics, polarization, and directional migration events that drive neutrophil chemotaxis. Inhibition of fMLP-stimulated neutrophil chemotaxis by *T. denticola* Msp was reported to involve inhibition of Rac1 activation and localization [30], but mechanisms leading to Rac1 inhibition were not clear. Novel findings of the present study indicate that cell exposure to Msp probably causes a decrease in the effective local concentration of PIP3 upon fMLP-stimulation, through a combination of the inhibition of PI3-kinase activity and the activation of lipid phosphatase PTEN. The decreased PIP3 concentration would 1) fail to recruit and activate Akt; 2) fail to activate Rac1 through the well-recognized positive feedback loop, which in turn would 3) diminish the uncapping of actin filaments that is required for *de novo* actin assembly at the leading edge, which ultimately would 4) inhibit functional cell polarization and chemotaxis. A major finding of this study is the crucial role of lipid phosphatases, as experimental inhibition of PTEN activity reversed all of the inhibitory effects of Msp listed above.

We have previously shown that Msp impacts phosphoinositide production and recruitment via lipid phosphatase activity in fibroblasts, as a novel mechanism leading to actin rearrangement [34]. In the present study, using the combination of chemical inhibition together with specific immunoprecipitation assays, we confirm PTEN as the lipid phosphatase that is activated when neutrophils are exposed to Msp.

A recently proposed model predicts that PTEN contributes negatively to the regulation of directional sensing in neutrophils by blocking the development and maintenance of the PIP3 gradient at the leading edge [6]. Yet, a direct role for PTEN in neutrophil chemotaxis is controversial, depending on the cell type and assay system used [6], [16], [17], [20], [38]. In fact, neutrophils treated with the PTEN inhibitor VO-OHpic are still able to migrate effectively towards fMLP [6]. Our new data indicate that Msp upsets the regulation of neutrophil directionality through activation of lipid phosphatase PTEN by disruption of the PIP3 balance that is crucial for establishing the neutrophil's directional “compass”. Indeed, our findings complement previous reports that PTEN may act as a negative regulator of many neutrophil functions, including actin polymerization and sensitivity to chemoattractants [17], [20].

It is well established that PI3-kinase and PTEN work in opposing fashion in cells, including neutrophils, to regulate phosphoinositide metabolism. PI3-kinase activity has been shown to be required for the initial polarization and chemotaxis response of neutrophils to fMLP [15]. Coincident with its activation of PTEN, Msp decreases PI3-kinase activity, which probably adds to its disruption of normal phosphoinositide metabolism downstream of fMLP stimulation in neutrophils. We found evidence of substantially reduced PIP3 signaling and obvious defects in neutrophil polarization and chemotaxis. A previous report that neutrophils lacking PI3-kinase may still migrate effectively [38], raises the significance of our finding of coincident activation of PTEN by Msp.

It is clear that Msp is able to effectively inhibit phosphoinositide signaling and Rac1 activation in neutrophils. Yet, other small GTPases such as Rac2 and Cdc42 are still activated normally following fMLP stimulation [30], suggesting that the defect is not solely at the level of chemoattractant sensing and initial signal development. Thus, it is likely that Msp acts by affecting the local PIP3 levels and/or the effective concentration of PIP3 required for Rac1 activation through a positive feedback loop [39]. We have previously reported that Msp causes mislocalization of the PH-AKT-RFP probe, which highlights spatial localization of PI3-kinase products, in response to fMLP stimulation, with multiple punctate distribution points rather than concentration at the leading edge [30]. These findings are also reminiscent of data reported for neutrophils lacking PI3-kinase-γ. When exposed to fMLP, these cells showed random distribution of actin and loss of co-localization with Akt due to the inability to stabilize and maintain the leading edge [14]. Taken together with new Akt immunofluorescence data, presented herein, this suggests that PIP3 production and localization to achieve a critical concentration at the leading edge is effectively disrupted by Msp treatment.

Our group has recently shown in neutrophils that different Rac isoforms localize to specific membrane domains based on alterations in phospholipid membrane composition. Specifically, during chemotactic leading edge formation, accumulation of PIP3, concomitant with an increase in negative charge, recruits Rac1 to the plasma membrane [40]. Thus, alterations in PIP3 production induced by Msp probably result in diminished Rac1 localization and activation following fMLP stimulation. In fact, treatment of neutrophils with the PI3-kinase inhibitor wortmannin, prevented accumulation of a charge biosensor at the leading edge, confirming the importance of PIP3 production in the development of a charge gradient during chemotaxis [40].

Exposure of neutrophils to Msp has been reported to prevent actin assembly at free barbed ends in response to fMLP stimulation [31]. This would be expected to affect the protrusion of the leading lamellipodium and subsequent migration. Rac1 has been shown to be the primary mediator of actin uncapping-dependent free-barbed end formation in fMLP activated neutrophils [35]. While we recognize that other actin polymerization pathways may conceivably be impacted by Msp, we have found conclusive evidence that Msp diminishes the release of fMLP-stimulated actin uncapping proteins gelsolin and CapZ. The effect of Msp was reversed by 1) chemical inhibition of PTEN and 2) transduction of a constitutively active Rac1.

The finding that cell exposure to Msp decreased the extent of actin-uncapping in fMLP-stimulated neutrophils is contrary to our previous observations of Msp-treated fibroblasts [34]. Yet, the activation states of the cells in these studies need to be considered. Our fibroblasts were “resting” cells, and most of our Msp experiments in neutrophils concentrated on fMLP-activated cells. In fact, Msp exposure alone, without subsequent fMLP stimulation, increased actin uncapping in neutrophils in a similar manner to fibroblasts (data not shown), suggesting a common mechanism. The ability of PTEN inhibition to increase PIP3 production and subsequent Rac1 activation together with the fact that transduction of active Rac1 alone prevented the Msp-mediated impairment of actin uncapping in neutrophils, suggests that Msp acts primarily at the level of PIP3 in the hierarchical feedback loop of PIP3 and Rac1 activation.

PTEN can be regulated at multiple levels including transcription, post-translational as well as through changes in localization [41], [42]. While it does not appear that Msp induces PTEN activation through changes in phosphorylation levels (data not shown), it has been reported that PTEN lipid phosphatase activity is enhanced by PtdIns[(4], [5)]P_2_ through changes in localization as well as conformational changes [43], [44]. This is an intriguing possibility to consider as we show in this study that Msp induces changes in cellular phosphoinositide levels in neutrophils as well as fibroblasts which we have reported earlier [34]. Likewise, Msp causes local recruitment of PtdIns[(4], [5)]P_\_ to the plasma membrane in fibroblasts [34] which could play a role in recruiting and activating lipid phosphatases [45].

Though other bacterial pathogens have been reported to upset the lipid phosphatase activity of target cells, the key mechanism is often mediated by a toxic export protein injected via a transmembrane secretion system. There is no evidence of a type III or analogous secretion system in *T. denticola* [46]. Native Msp is a large protein complex of the outer sheath with function and topology hypothesized to be similar to pore-forming proteins of other Gram-negative bacterial pathogens [47]. Msp is cytotoxic to some cells such as epithelial cells through porin channel formation and membrane depolarization [48], [49]. In cells such as fibroblasts and neutrophils, Msp most likely mediates its effects on host cell signal transduction pathways upon contact with its plasma membrane. Msp does not appear to induce significant permeability of the cell membrane or loss of viability in neutrophils [31] and remains extracellular or associated near the plasma membrane [33]. In fibroblasts, Msp induces calcium ion influx within a few seconds of exposure and inhibits cytosolic Ca2+ transients in neutrophils stimulated by fMLP [31], [33]. It is possible that Msp acts upon a specific host cell receptor(s) to mediate its effects. For example, it has been reported that Msp interacts with at least one cell surface protein of 65-kDa in HeLa epithelial cells [49], although this protein or other putative receptors have yet to be identified in neutrophils or characterized further. Many bacterial protein toxins are able to interfere with host lipid metabolism including modifying the activity of lipid phosphatases and kinases [50]. Our findings presented here extend this knowledge and indicate that Msp is a novel protein that can modulate activity of PI3-kinase and the lipid phosphatase PTEN, as a pathogenic mechanism to modulate downstream cell-signaling pathways leading to impairment of crucial host cell functions.

In summary, exposure of neutrophils to *T. denticola* Msp upsets the phosphoinositide balance that normally drives polarization and chemotaxis through coincident activation of PTEN and inhibition of PI3-kinase activation. The ability of Msp to diminish PIP3 levels leads to hierarchical inhibition of downstream chemotactic events, including Rac1 activation, actin filament uncapping, polarization, and directed cell migration.
